# Clinical, ultrasonographic, and histopathologic findings in seven horses with Descemet's membrane detachment: A case series

**DOI:** 10.1111/vop.12710

**Published:** 2019-09-22

**Authors:** Inge J. M. Slenter, Hanneke Hermans, Jos M. Ensink, Dorien S. Willems, Stefanie Veraa, Guy C. M. Grinwis, Michael H. Boevé

**Affiliations:** ^1^ Faculty of Veterinary Medicine Department of Clinical Sciences of Companion Animals, Ophthalmology Section Utrecht University Utrecht The Netherlands; ^2^ Faculty of Veterinary Medicine Department of Equine Sciences, Surgery Section Utrecht University Utrecht The Netherlands; ^3^ Faculty of Veterinary Medicine Department of Clinical Sciences of Companion Animals Diagnostic Imaging Utrecht University Utrecht The Netherlands; ^4^ Faculty of Veterinary Medicine Department of Pathobiology Utrecht University Utrecht The Netherlands

**Keywords:** bullous keratopathy, corneal edema, Descemet's membrane, equine, glaucoma, ultrasound

## Abstract

**Objective:**

To describe ultrasonography as a diagnostic method of in vivo Descemet's membrane detachment (DMD) in horses. Animals studied: Seven horses (three Icelandic horses, two Dutch Warmblood horses, one Appaloosa, and one Welsh Pony), presenting with moderate‐to‐severe focal or diffuse corneal edema, in whom DMD was suspected on ultrasonographic examination and confirmed with histopathology, were studied.

**Procedure:**

A retrospective analysis of case records of horses with suspected DMD was performed.

**Results:**

Median age at presentation was 14 years (range 11‐24). Clinical signs in eyes with DMD were unilateral in all horses and included blepharospasm and epiphora (6/7), buphthalmos (5/7), moderate‐to‐severe focal or diffuse corneal edema (7/7), corneal epithelial bullae (4/7), corneal neovascularization (4/7), Haab's striae (2/7), corneal endothelial precipitates (1/7), fibrin in the anterior chamber (1/7), focal cataract (2/7), and pigment deposits on the anterior lens capsule (1/7). During transpalpebral ultrasonography, a distinct linear echogenic structure was noted in the anterior chamber, initially diverging from, and later running parallel to, the posterior lining of the cornea in all eyes studied. In all cases, the cornea was severely thickened and echogenic, consistent with edema, and DMD was suspected. In all horses, the clinical signs progressed and the affected eye was eventually enucleated. Histopathology revealed DMD (7/7), spindle cell proliferation (4/7), Descemet's membrane reformation (3/7), and inflammation of the anterior uvea (5/7). Overall incidence was 1.04%.

**Conclusions:**

Ultrasonography is an adequate tool in diagnosing DMD in horses. Descemet's membrane detachment should be included in the differential diagnosis in horses with dense focal or diffuse corneal edema.

## 1 INTRODUCTION

Descemet's membrane (DM) is the basement membrane produced by the corneal endothelium. It is a homogenous acellular membrane containing various types of collagen but mainly Types III, IV, and VIII.^1^, ^2^ The average thickness of DM in the adult horse measures 38 µm, increasing with age at a rate of 2 µm/year as it is formed throughout life.^3^ Descemet's membrane has been described to have certain elastic properties in the horse.^2^ In the normal equine eye, DM is under slight tension, and in the event of a rupture, DM tends to curl like a scroll.^1^, ^2^, ^4^ A tear in DM, such as that created by a corneal incision or trauma to the DM during ocular surgery, can result in aqueous humor flowing in between the DM and the adjacent posterior part of the corneal stroma, leading to a DM detachment (DMD).^5^ In the case of DMD, the pump mechanism responsible for preventing uncontrolled fluid influx into the corneal stroma, located within the corneal endothelium, is displaced. As a result, severe corneal edema, irreversible bullous keratopathy, recurring corneal ulcers, and, ultimately, loss of functional vision may occur.^4^, ^6^, ^7^, ^8^ Descemet's membrane detachments have been reported in human literature in association with various corneal and intraocular surgeries.^6^, ^7^, ^9^, ^10^ Recently, DMD has also been described in horses that underwent cataract surgery^11^ and in horses with glaucoma.^8^

Several diagnostic modalities for visualization of a DMD have been documented in human literature. In clear corneas, DMD can be observed with slit‐lamp biomicroscopy and gonioscopy.^5^, ^12^ In cases with substantial corneal edema, the details of the detached DM are optically obscured. Hence, other diagnostic procedures have been recommended, including ultrasound biomicroscopy (UBM), specular and confocal microscopy, and anterior segment optical coherence tomography (OCT).^5^, ^12^, ^13^, ^14^, ^15^, ^16^ As described in literature, DMD in horses has been diagnosed intraoperatively, ultrasonographically,^11^ and on histopathology.^8^, ^11^ In one study, corneal spectral domain optical coherence tomography (SD‐OCT) revealed a partial detachment of DM and associated endothelium floating in the anterior chamber, in a horse with heterochromic iridocyclitis and secondary keratitis.^17^ To the authors’ knowledge, only one study reports ultrasonographic findings of a DMD in a horse.^11^ In this case, however, the DMD resolved, and therefore, no histopathology was performed that could confirm the diagnosis. The purpose of our study was to describe ultrasonographic findings of DMD in horses confirmed by histopathology.

## 2 MATERIALS AND METHODS

### 2.1 Case selection

Case records of horses that were presented to the Department of Equine Sciences of the Faculty of Veterinary Medicine, Utrecht University, The Netherlands, between January 2012 and August 2018, with suspected DMD on ultrasonography were reviewed. Only horses with confirmed DMD on histopathology were included in the present study. Breed, age at onset, clinical history, ophthalmic examination results, treatment, complications, and clinical and/or surgical outcome were recorded. A search of the database of the Pathology Department was performed using “Descemet's membrane” or “Descemet's membrane detachment” to possibly identify additional cases.

### 2.2 Ophthalmic examination

All horses were subjected to a complete ophthalmic examination of both eyes. Ophthalmic examinations were performed by an equine ECVS diplomate (HH/JE, n = 7), and/or a board‐certified (ECVO) veterinary ophthalmologist (MB, n = 7), and a resident in veterinary ophthalmology (IS, n = 4), and included neuro‐ophthalmic testing, hand‐held slit‐lamp biomicroscopy (SL‐15, Kowa Optimed, Inc), tonometry (TonoVet, Icare^®^ Finland Oy) for all cases except for case 3 at the time of presentation for suspected DMD, fluorescein staining (BioGlo BIO FLUORO, BIOTECH) and direct ophthalmoscopy (Pneumatic Otoscope Welch Allyn, Skaneateles falls NY 13153 USA). If necessary patients were sedated with detomidine hydrochloride (Domosedan, Orion Corporation, Espoo, Finland), 0.01 mg/kg bodyweight iv ocular hypertension was defined as an increase in intraocular pressure (IOP) above 30 mm Hg.^18^

### 2.3 Ultrasonography

All ultrasonographic examinations were performed by a resident in veterinary diagnostic imaging (DW) and/or a board‐certified (ECVDI) veterinary radiologist (SV) and included imaging of the eye and retrobulbar area. Both eyes were examined. Standard transpalpebral ultrasonography of the equine globe was performed using a linear transducer in combination with a curvilinear transducer set between 8 and 12 MHz (PHILIPS C8‐5, PHILIPS L12‐3 PHILIPS L15‐7io HD11 XE, and Epiq 5, Philips healthcare, Eindhoven, The Netherlands). The frequency applied was dependent on the depth of the ocular structure to be examined. Highest available frequency was applied for the cornea and anterior chamber. Mechanical and thermal indices were ranging between 0.7 and 0.9. If necessary, patients were sedated as described for “ophthalmic examination.”

A small overlap exists between the range of reported ultrasonographic axial globe lengths for buphthalmic equine globes (40.0‐52.5 mm, mean 46.3 mm)^19^ and ultrasonographic axial globe lengths documented in the nonbuphthalmic horse (37.5‐42.2 mm).^20^, ^21^, ^22^, ^23^, ^24^ For this study, we therefore decided to determine an eye to be buphthalmic if the ultrasonographic axial globe length was equal to, or exceeded 45 mm, or if the increase in axial globe length, in comparison with the contralateral globe, was more than could be explained by the increase in corneal thickness due to corneal edema. We chose this cut‐off value based on the highest reported ultrasonographic axial globe length (42.2 mm) documented in the nonbuphthalmic horse and taking increase in corneal thickness due to corneal edema into account as a potential factor for an increased ultrasonographic axial globe length.

The corneal thickness was measured from the surface epithelium until the hyperechoic line representing the endothelial lining of the cornea in both eyes. At the region of the DMD, the corneal thickness was measured from the surface epithelium until the posterior margin of the corneal stroma (Figure 2B, measurement depicted with a double arrow). In cases with focal corneal edema, the corneal thickness was compared with clear cornea in the same eye and with the corneal thickness of the contralateral healthy eye. In cases with diffuse corneal edema, the corneal thickness was compared with the corneal thickness of the contralateral healthy eye. As case 7 presented with bilateral ophthalmic disease, the corneal thickness of the DMD affected eye was compared with the corneal thickness of the healthy globes of the other Icelandic horses.

### 2.4 Histopathology

The enucleated globes were fixed in either 10% neutral buffered formalin or Davidson's fixative solution. The globes were routinely processed for histopathology and stained with hematoxylin and eosin for evaluation of the general morphological features of the eye, and alcian blue and periodic acid Schiff stains for visualization of the extracellular matrix and Descemet's membrane, respectively. All samples were examined by a board‐certified veterinary pathologist (GG). Descemet's membrane detachment was defined as the separation of the DM from the posterior corneal stroma in the presence of spindle cell formation and/or flattened endothelial cells and reformation of the DM.

### 2.5 Data analysis

The collected data are presented descriptively, with median and ranges included where appropriate. Incidence rates were calculated by dividing the number of new DMD cases per time period, with the total number of primary ophthalmic consultations at our facility in the respective time period.

## 3 RESULTS

Nine horses were identified with suspected DMD on ultrasonography, and two cases were excluded from this study as histopathology was not available (one case was enucleated, but no histopathology was performed, and the other case improved with topical therapy). During the time of the study, 671 new ophthalmic cases were presented to the Equine Department, making the overall incidence 1.04% (7/671) for all confirmed DMD cases and 1.34% (9/671) for confirmed and suspected cases combined. Higher incidence rates for suspected and confirmed cases combined were seen in 2017 (4/138; 2.90%) and 2018 (2/101; 1.98%).

### 3.1 Signalment

The horses included in this study consisted of four geldings and three mares. The median age of the horses was 14 years (11‐24 years). Breeds included three Icelandic horses, two Dutch Warmblood horses, one Welsh pony, and one Appaloosa.

### 3.2 Ophthalmic examination

**Figure 1:**
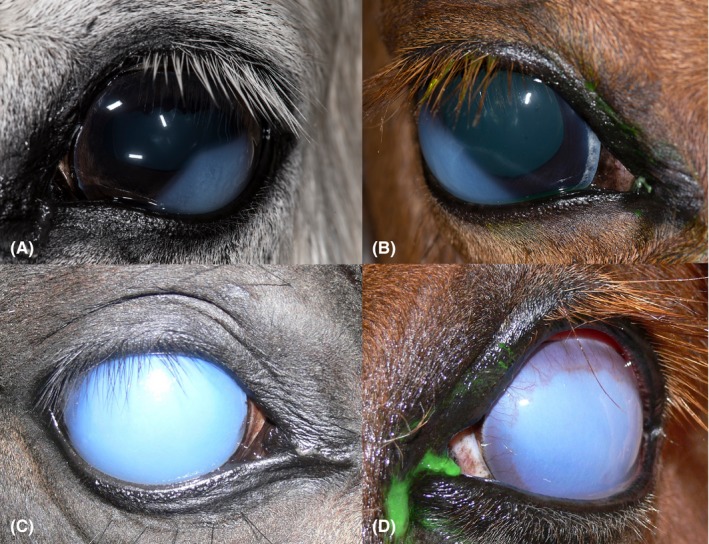
Focal (A and B) corneal edema in cases 1 and 2, respectively, and severe diffuse corneal edema (C and D) in cases 5 and 6, respectively, presenting with DMD. Peripheral neovascularization is also visible in panel D

The clinical presentation, including the signalment, of the different cases is summarized in Table 1. All cases presented with unilateral corneal edema. Five cases initially presented with focal corneal edema (Figure 1A and B), at time of presentation to our clinic. Cases four and five presented with complete diffuse corneal edema (Figure 1C and D). Four of the five cases with initial focal corneal edema eventually progressed into complete diffuse corneal edema over a period of 1 week to 5 months.

**Table 1:** Signalment and clinical presentation of seven horses with DMD

Case	1	2	3	4	5	6	7
Breed	Icelandic horse	Welsh pony	Dutch Warmblood	Appaloosa	Dutch Warmblood	Icelandic horse	Icelandic horse
Gender	Gelding	Mare	Mare	Mare	Gelding	Gelding	Gelding
Age of onset (years)	13	13	14	11	24	17	15
Weight (kg)	416	403	570	616	615	386	330
Ophthalmic examination							
Affected eye	OS	OD	OS	OD	OD	OS	OD
Discomfort (blepharospasm/epiphora)	+	+/‐	+	‐	+/‐	+	+/‐
lOP (mm Hg) Non‐DMD‐affected eye	19	15	N/A	17	24	29	30
lOP (mm Hg) DMD‐affected eye	26	25	N/A	18	29	21	80
Buphthalmos^a^			X	X	X	X	X
Focal corneal edema	X	X	X			X	X
Diffuse corneal edema		X	X	X	X	X	X
Bullous keratopathy	X		X	X	X		
Haab's striae		X	X				
Perilimbal vascular ingrowth			X	X	X	X	
Keratic precipitates				X			
Cataract		X					X
Lens luxation							X
Fibrin/pigment anterior lens capsule		X					

Ultrasonographic examination	DMD affected	Non‐DMD affected	DMD affected	Non‐DMD affected	DMD affected	Non‐DMD affected	DMD affected	Non‐DMD affected	DMD affected	Non‐DMD affected	DMD affected	Non ‐DMD affected	DMD affected	Non‐DMD affected
Axial globe length (mm)	38	38	39^b^	37	45	41	42	38	48	42	46	39	45	45
Clear corneal thickness (mm)	0.9	0.9	1	1		0.9		0.9		1		1		1.3
Edematous corneal thickness (mm)^c^	2.3		2.5		3.1		2.5		3		2.5		1.9	

Notes

OD, right eye; OS, left eye; +, discomfort, +/‐, moderate discomfort, ‐, no discomfort.

^a^ Buphthalmos was defined as an axial globe length equal to or exceeding 45 mm in case numbers: three, five, six, and seven or if the increase in axial globe length in comparison with the contralateral healthy eye was more than could be explained by the increase in corneal thickness due to corneal edema in case number: four.

^b^ There was a clinical impression of buphthalmos, which was not appreciable on ultrasonography.

^c^ For the DMD affected eyes with focal corneal edema, corneal thickness was measured at the area of the edema.

Four cases (cases 1, 3‐5) subsequently developed epithelial bullae formation and became fluorescein positive. These cases had associated ocular discomfort (ie, blepharospasm and/or epiphora). Case three was previously presented for recurring episodes of anterior uveitis, and case two revealed intraocular sequelae upon ophthalmic examination at our clinic, indicative of previous inflammatory episodes (ie, focal cataract and fibrin, as well as pigment deposits on the anterior lens capsule). Case four did not present nor develop signs of ocular discomfort. Corneal cytology was performed in case one and revealed normal epithelial cells and few inflammatory cells. Case seven presented with absent menace responses OU, negative direct pupillary light reflex (PLR) OS and negative consensual PLR from OD to OS (due to corneal edema OD the pupil could not be visualized), negative dazzle reflexes OU, bilateral anterior lens luxation, buphthalmos, and focal cataract. After consultation, the lens relocated posterior from the iris in the right eye. Only the right eye presented with extensive focal corneal edema, which progressed to dense diffuse corneal edema. There were otherwise no clinical signs of inflammation, and however, further diagnostic testing (eg, aqueocentesis and serology) was declined by the owner.

Measurements of the IOP were available for six of the seven cases. One of the six horses undergoing tonometry presented with ocular hypertension. The IOP for case three was not available at time of presentation of suspected DMD. The horse was previously presented for equine recurrent uveitis, at which point the IOP was 27 mm Hg OU.

Cases two and three also showed Haab's striae associated with stretching of the bulbus, and four cases showed perilimbal deep corneal neovascularization (cases 3‐6) suggestive of primary corneal inflammation, intraocular inflammation (uveitis), and/or glaucoma.

In cases presented with extensive focal corneal edema (cases 3, 6 and 7) and dense diffuse corneal edema (cases 4 and 5) visualization, the posterior segment was not possible. In case 1, neuro‐ophthalmic examinations were normal and no fundus abnormalities were noted. Case 2 had a normal fundic examination and was visual at initial presentation, but as the edema progressed the pony lost its menace response and the dazzle and consensual pupillary light reflex. The remaining cases (cases 3‐7) had absent consensual PLRs, absent menace responses, and dazzle reflexes.

According to the provided medical history, no (intra)ocular surgeries had been performed previously on any of the cases, except for case three. Case three had been treated with a suprachoroidal cyclosporine implant for equine recurrent uveitis a year prior to presentation for suspected DMD.

Additional treatment options, such as modified Gundersen inlay flaps, or the use of topical hyperosmotics were discussed with the owners. One case (case 6) was additionally treated with sodium chloride hypertonicity ophthalmic ointment 5% q3h, but did not improve clinically. None of the owners elected surgical intervention. Ultimately, all owners decided to enucleate affected eye because of persistence or progression of the clinical signs despite medical therapy and poor prognosis regarding vision. Because of bilateral blinding disease, the owner of case 7 elected euthanasia. Corneal edema had existed for 6‐163 days prior to enucleation.

### 3.3 Ultrasonography

In all eyes examined, severe corneal thickening (1.9‐3.1 mm) with a diffusely increased echogenicity was noted, consistent with corneal edema. At the time of ultrasonographic examination, the corneal edema was focal in two cases (cases 1 and 2) and diffuses in five cases (cases 3‐7).

**Figure 2:**
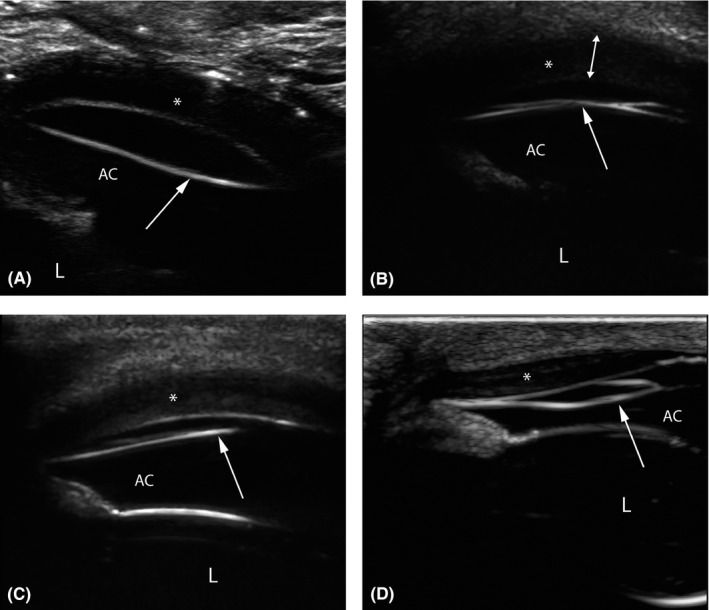
Ultrasonographic images of cases 6 (A), 2 (B), and 3 (C and D) showing a thin, well‐defined hyperechoic linear structure (white arrow) deviating from the posterior stroma (*) toward the iris. L, lens; AC, anterior chamber. The double‐headed arrow in panel b represents the corneal thickness in an area of Descemet's membrane detachment

All cases showed a thin, well‐defined hyperechoic line in the anterior chamber (Figure 2A‐D). This line, presumably detached DM and associated endothelium, deviated from the posterior stroma, extending into the anterior chamber, creating a space between these two layers. At first, running partially oblique and further mostly parallel to the posterior stroma. In five cases (cases 3‐7), buphthalmos was present. In case seven, the buphthalmos was bilateral. Measurements of the axial globe length and corneal thickness for both eyes are presented in Table 1.

Additional findings in the ultrasonographic examination of the eyes included echogenic regions in the lens consistent with cataract (n = 2 eyes; cases 2 and 7), lens luxation (n = 2 eyes; case 7), echogenic lines or particles in the vitreous (n = 7 eyes; cases 2 and 7 OU, cases 3‐5 only the affected eye), and thickened sclera/chorioretina (n = 1 eye; case 5).

### 3.4 Histopathology

**Figure 3:**
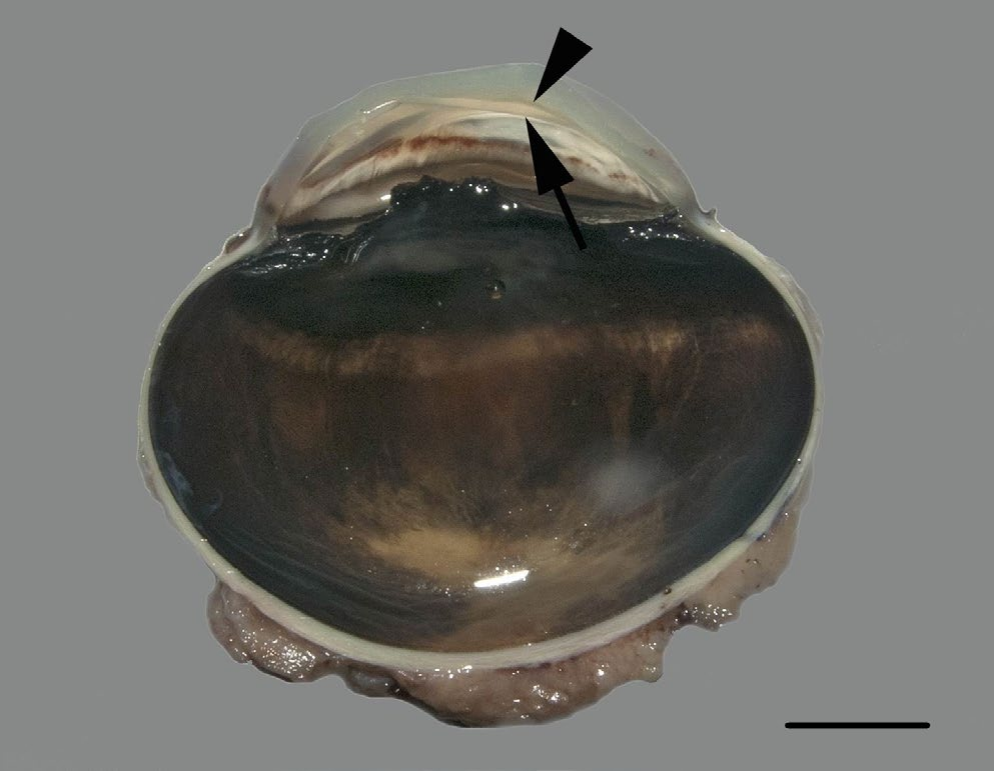
Macroscopic image of a cross‐section of the globe of case 3 revealing DMD (black arrowheads). Bar = 1 cm

**Figure 4:**
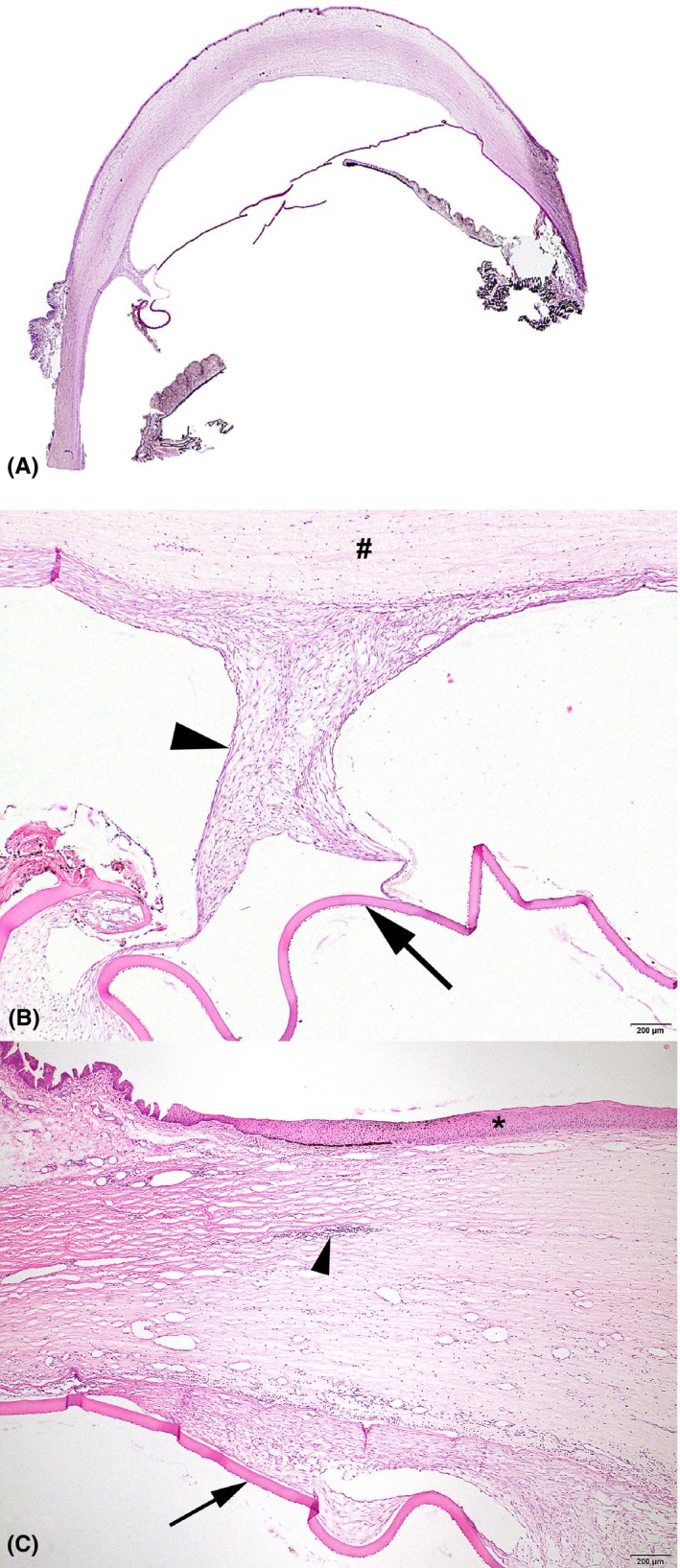
A, Direct scan of tissue slide of the globe of case 6. Descemet's membrane detachment is appreciated over a large area of the corneal stroma. B, Spindle cell proliferation (black arrowhead) is seen on the corneal stromal (^#^) side of the DMD (black arrows) and attached to the DM. C, At the area of DMD (black arrows), there is an inflammatory infiltrate in the corneal stroma (black arrow head). The corneal epithelium is marked with a black asterisk. Image a is a hematoxylin and eosin staining. Images b and c are periodic acid of Schiff (PAS) staining

Figure 3 illustrates the DMD macroscopically (case three). In cases six and seven, DM appeared to be almost completely detached over the entire length of the cornea (Figure 4A). Other cases showed variable lengths of DMD. At the area of the detachment, DM showed focal ruptures in most cases either due to processing of the globe or due to in vivo ruptures possibly associated with DMD. Moderate to profound spindle cell proliferation between the DMD and the corneal stroma (Figure 4B and C) was observed in four globes (cases 3, 4 and 6‐7). In the remaining globes, flattened endothelial cells and reformation of the DM (newly formed basement membrane material) could be appreciated in the area adjacent to ruptured DMD, presumably in an effort to re‐establish normal anatomical and physiological configuration. These findings were either with or without mild spindle cell proliferation present. In five cases (cases 1‐4 and 6), a moderate to profound lymphoplasmacytic inflammation of the iris and/or ciliary body was observed consistent with anterior uveitis and HIK.^17^, ^25^ Collapse of the iridocorneal angle was observed in case four. Posterior segment evaluation was only performed in two cases (cases 1 and 5). Case 1 showed some degenerative changes in the optic disk including the presence of several macrophages containing a little hemosiderin and some lipofuscin pigment but the adjacent retina was not clearly atrophic. Case 5 showed extensive cupping of the optic disk, and however, the retina could not be evaluated because it detached during fixation and was not present in the tissue section. No additional DMD cases were identified when searching the database for case records of the pathology department.

## 4 DISCUSSION

To the authors' knowledge, this is the first report describing ultrasonographic findings of DMD in horses, including confirmation of the diagnosis by histopathology. The findings of this study confirm the importance of an ultrasonographic examination, with special attention to the cornea and anterior chamber, as part of a standard ophthalmological work‐up in equine eyes with corneal edema.

The clinical signs described in the present study are in agreement with previous studies concerning DMD in horses,^8^, ^11^ with dense focal and diffuse corneal edema being the most consistent findings. The former studies described DMD in association with glaucoma^8^ and cataract surgery.^11^ In the present study, none of the affected eyes had undergone cataract surgery. In contrast to the study of Henriksen 2017 et al,^8^ only one of the horses in our study presented with an increased intraocular pressure and associated buphthalmos. However, four additional cases presented with buphthalmos of the affected eye on admission, indicating globe distention most commonly associated with chronic glaucoma.^26^ Furthermore, on histopathology case 4 revealed a collapsed iridocorneal angle probably secondary to chronic anterior uveal inflammation and case 5 showed cupping of the optic disk, both consistent with glaucoma.^26^ In contrast, Haab's striae were relatively uncommon in our cases. However, some cases may have gone unrecognized because of an inability to appropriately asses the cornea due to dense corneal edema.

Six cases presented with mild‐to‐moderate blepharospasm and epiphora. We associated these signs with ocular discomfort due to either an underlying primary ocular disease (eg, uveitis) or, subsequently, due to the bullous keratopathy and corneal ulcerations that resulted from the chronic corneal edema and/or glaucoma.

The median age of the horses in this study was 14 years, which is considerably lower than the median age of 22 years in the study by Henriksen et al.^8^ The difference in age between the two studies could reflect different etiologies of DMD or may be due to the relatively low number of cases in both studies. The findings of both studies suggest that DMD is more likely to occur in middle‐aged to older horses.

The pathogenesis of DMD in horses is presently unknown. Based on previous reports and the findings of our study, there are multiple plausible causes and it is possibly multifactorial in some cases. Reports in human literature support this hypothesis as DMD in humans has not only been associated with various intraocular surgeries,^6^, ^7^ but has also been described to occur years after ocular surgery,^27^, ^28^, ^29^ after sodium cyanide chemical injury,^12^ and in an infant after a forceps‐related birth injury.^30^ In humans, the possibility of a pre‐existing anatomic predisposition to DMD has been explored and suggested an abnormality in the fibrillary stromal attachment to DM.^31^ Mutations in transforming growth factor β (TGF‐β) induced gene results in a weak adhesion of DM to the stroma as a consequence of a TGF‐β‐induced protein dysfunction.^10^

In the present case series, there were two horses with signs of (pre‐existing) anterior uveitis noted upon initial ophthalmic examination. Histopathology confirmed ocular inflammation in these cases and revealed three additional cases with minor‐to‐moderate inflammation in the iris and/or ciliary body. In human literature, pre‐existing endothelial disease was found to be a significant preoperative risk factor associated with DMD after cataract surgery.^32^ In horses, inflammatory cells and associated components released into the anterior chamber during equine intraocular inflammatory diseases, like equine recurrent uveitis (ERU) and heterochromic iridocyclitis and keratitis (HIK), may result in endothelial damage.^17^, ^33^ Further research is necessary to determine the effects of these intraocular inflammatory diseases on the endothelial cells and DM in horses and whether they would form a risk factor for developing DMD. As ERU is relatively common in our equine population^34^ and DMD is rarely described, it is interesting to further investigate risk factors and to compare compositions of DM between diseased and healthy individuals, in relation to age.

Although the equine DM is likely to possess elastic properties,^1^, ^2^, ^4^ the tension on DM due to stretching of the globe, in five of our cases presented with buphthalmos, may have exceeded its elasticity and, therefore, resulted in a tear in DM that might have predisposed to DMD. As far as the authors know, DMD has not been described in relation with (over)stretching of the globe and further research on the biomechanical properties of DM is necessary before a conclusive relationship between the two (DMD and stretching of the globe) can be established.

Descemet's membrane detachment on histopathologic examinations can be artifactual due to processing ((formalin) fixation, paraffin embedding, and sectioning) of the enucleated globe. However, when spindle cell proliferation is present near the exposed corneal stroma, it helps in determining a clinical DMD (D. Dubielzig, personal communication, May 22, 2018). Spindle cell proliferation was a common finding in our study, however, in contrast to former studies,^8^, ^11^ not apparent or as elaborate in all eyes. Topical corticosteroids may have inhibited fibroblastic activity.^35^ As far as the authors know, it is not known how long it takes for spindle cell formation to occur in horses once DMD occurs. Time between onset of symptoms and enucleation could have possibly been too short for spindle cell formation to occur in some of our cases. The absence of spindle cell proliferation on histopathology in DMD has also been reported in one human case.^36^ The extensiveness of DMD might also influence the fibroblastic response as spindle cell proliferation appeared most elaborate in cases with large detachments. Retrocorneal spindle cell proliferation is also a common finding in horses with HIK,^17^ further supporting the exploration of a possible relationship between this inflammatory condition and DMD in horses.

Limitations of the current study are the retrospective nature of the study design, lack of a control group, and the small sample size. Furthermore, a major limitation of the current study is the lack of detailed posterior segment evaluation. Especially as six of our seven cases lost PLRs and dazzle reflexes, which indicates a loss of retinal function and/or optic neuropathy. All owners from the horses in the present case study, except for the owners from case 1, declined histopathologic examination. However, we decided to send the samples of the remaining cases to our pathology department to see whether our ultrasonographic suspicion of DMD could be confirmed with histopathology. The eyes of horses were too large to encompass all parts of the globe within the paraffin blocks and were therefore hemidissected with only the anterior segments being processed and evaluated. Although ocular hypertension is a major contributing factor in glaucoma, vision can deteriorate with normal IOPs,^26^ possibly explaining the negative PLRs and dazzle reflexes in five of our cases. Unfortunately, we cannot confirm the suspicion of chronic glaucoma nor the presence of retinal degeneration in six cases. We suggest detailed histopathologic posterior segment evaluation in future studies, as it could possibly aid in studying the etiology, pathogenesis, course of the disease process, and prognosis of DMD in horses. Another limitation of our study, not uncommon in retrospective case series, is the lack of the IOP documentation at time of DMD presentation of case 3. It is unknown why these data were not documented in the patient file. However, as case 3 met our inclusion criteria (DMD suspicion on ultrasonography and confirmed DMD on histopathology) we decided to include this case in our study. Even though diagnostic imaging was performed at a rather low resolution, we did not see any DMD cases confirmed with histopathology that were missed by ultrasonography. Ultrasound biomicroscopy, specular and confocal microscopy, and anterior segment OCT are diagnostic modalities with higher resolutions and provide greater detail of the cornea and the anterior segment in comparison with ultrasonography. Nevertheless, although becoming increasingly available, not all clinics may have access to these more advanced diagnostic imaging techniques. As ultrasonography is a readily available tool in almost all equine practices, it provides a good alternative and, based on our study, is sufficiently adequate in imaging DMD. Awareness of the possibility of a DMD will increase the chance of finding one on ultrasound as we demonstrate an increase in incidence in most recent years.

In conclusion, ultrasonography is confirmed to be a useful and noninvasive diagnostic modality in evaluating the posterior part of the cornea for DMD in equine cases presenting with unilateral diffuse or focal corneal edema. Descemet's membrane detachment is increasingly being recognized and should be considered as a differential diagnosis in horses with dense focal and diffuse corneal edema, especially in otherwise salvageable globes as it has the potential to progress. Further studies are necessary to identify risk factors and treatment options.
